# Tumor-derived exosomal miRNA-141 promote angiogenesis and malignant progression of lung cancer by targeting growth arrest-specific homeobox gene (GAX)

**DOI:** 10.1080/21655979.2021.1886771

**Published:** 2021-02-25

**Authors:** Wulong Wang, Guodai Hong, Siyuan Wang, Wenbin Gao, Ping Wang

**Affiliations:** a Department of Radiotherapy, Tianjin Medical University Cancer Institute and Hospital, National Clinical Research Center for Cancer, Tianjin Key Laboratory of Cancer Prevention and Therapy, Tianjin’s Clinical Research Center for Cancer, Tianjin, China; b Department of Oncology, The Second Affiliated Hospital of Baotou Medical College, BaoTou, Inner Mongolia, China; c Department of Oncology, The Third Affiliated Hospital of Shenzhen University, Shenzhen, Guangdong, China

**Keywords:** Lung cancer, exosome, miRNA-141, angiogenesis, GAX

## Abstract

Previous researches have suggested that exosomal miRNA-141 has association with metastatic lung cancer, however, its role and regulatory mechanism require further study. In this study, exosomes were isolated from lung cancer patients and normal human serum and identified. We found that the expression of miRNA-141 was up-regulated in the lung cancer serum exosomes compared with the normal serum exosomes. When the exosomes were extracted for co-culture with HUVECs, they were absorbed and distributed around the nucleus by confocal microscopy. Moreover, exosomal miRNA-141 from A549 significantly not only promoted the migration and invasion of A549 but also increased the cell proliferation, tube formation of HUVECs. In order to reveal the mechanism of exosomal miRNA-141, bioinformatics analysis revealed that miRNA-141 targeted the binding of Growth arrest-specific homeobox gene (GAX) in the 3ʹUTR region, and confirmed by MS2-RIP assay and dual-luciferase assay. Exosome miRNA-141 could down-regulate the expression of GAX. Taken together, our results demonstrate that tumor-derived exosomal miRNA-141 promote angiogenesis and malignant progression of lung cancer by targeting GAX. It provides a new possibility for the treatment of lung cancer.

## Graphical abstract

**Figure:**
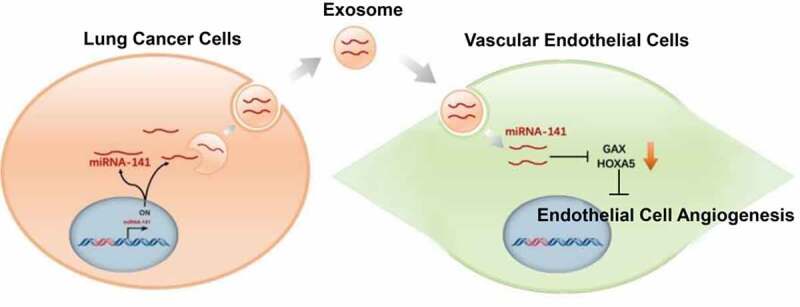

## Introduction

Lung cancer, as the leading cause of morbidity and mortality, cannot be neglected by human beings. The Global Cancer Report 2014 announced by the World Health Organization (WHO) demonstrated that lung cancer accounted for 19.4% of the total number of cancer deaths [1,2]. Lung cancer can be categorized into small cell lung cancer and non-small cell lung cancer in terms of the biological characteristics of it. The current treatment of lung cancer includes radiotherapy, combined chemotherapy based on platinum (such as cisplatin or carboplatin), and targeted drug therapy, in addition to surgical treatment. Due to the lack of knowledge of the etiology of the occurrence and development of lung cancer, the 5-year survival rate of patients does not exceed 15% [3–5]. Therefore, it is especially important to clarify the molecular mechanism of the occurrence and development of lung cancer, and then carry out targeted therapy based on this target, in order to improve the therapeutic effect, prolong the survival time and improve the life quality of patients who suffer lung cancer.

Exosomes are extensively found in urine, blood, saliva and other body fluids, and have good stability. Exosomes serve as an essential role in signal transmission between cells, and are important signal regulators whose functions can be affected by hormones and soluble growth factors [6]. In recent years, the research on exosomes and tumor progression has attracted more and more attention [7]. For example, studies have suggested that tumor cell-derived exosomes are rich in a variety of matrix metalloproteinases [8]. After being released into the extracellular matrix, they degrade the extracellular matrix and shear the adhesion molecules between cells, thus promoting the invasion and metastasis of tumor cells [9]. Alnedawik et al. found that EGFR-mutated gliomas could release exosomes rich in mutant receptor protein EGFR, endowing wild-type glioma cells with stronger invasion ability. According to most studies, exosomes accelerate tumor metastasis, mainly through the following four aspects: tumor cells directly open a pathway for tumor invasion and metastasis by secreting exosomes [8]. Tumor cells with high metastatic potential can increase both the invasion and metastasis ability of tumor cells themselves or other tumor cells with relatively low metastatic potential through exosomes [10]. Tumor microenvironmental intermediates and tumor cells communicate with each other through exosomes to promote tumor metastasis [10,11]. Tumor-derived exosomes are able to facilitate the distant colonization and proliferation of circulating tumor cells by transforming stromal cells in the microenvironment of distant metastasis [12,13]. Therefore, exosomes serve as a significant character in tumor metastasis and malignant progression, and are able to be markers and new therapeutic targets for predicting and preventing tumor progression [14].

MiRNAs, a type of endogenous single-stranded non-coding small RNA molecules having a length of 18–25 nucleotides are widely found in eukaryotic organisms. MiRNAs can selectively adhere to the three untranslated regions (UTRs) of mRNAs encoding protein-coding genes, which causes to degrade target mRNA and inhibiting post-transcriptional translation of target genes [15–17]. More and more studies have shown that there are high levels of miRNAs contained in exosomes, and exosomal miRNAs have been suggested to make a contribution to promote tumor proliferation and metastasis [18,19].

The high invasion and metastasis of lung cancer makes its death rate the first cause of death among all malignant tumors [18]. Behind this, what role does lung cancer cell-derived exosomes play? Does its exosome contain functional miRNAs? Is the focus of our attention. In this research, we focused on A549-derived exosomal miRNA-141 and studied its effect on A549 cells and HUVEC. We found that exosomal miRNA-141 could promote A549 migration and invasion, HUVEC proliferation and tube formation. Additionally, we confirmed that miRNA-141 could significantly inhibit the expression of target GAX.

## Materials and methods

### Exosome extraction and identification

Exosomes were separated through overspeed centrifugation. 0.25 mL of serum were taken and placed on ice, diluted with 11 mL PBS, and filtered with a 0.2 μm filter. Then the filtered serum was added to the centrifuge tube for 150,000 g and centrifuged overnight at 4 °C. After that, the exosomes at the bottom of the tube were washed with PBS to precipitate, and the exosomes were further centrifuged at a speed of 150,000 g at 4 °C for 2 h. Discard the supernatant, retain 200 μL solution at the bottom, and blow and mix the settling part.

The exosome solution suspended in PBS was mixed with dioxane acetate 1:1, and dropped on the copper network. After 1 min, filter paper was used to absorb the solution and dried and observed by electron microscope. The expressions of Alix and CD63 in exosomes were verified by western blot. Besides, the diameter of exosomes was gauged by the Zeta View method.

### High throughput analysis

From lung cancer patients and normal human serum exosomes, a total of RNA was extracted. And the RNAs were processed for construction as previously described [20]. The library was quantified on an Agilent 2100 (USA) and sequenced on an Illumina HiSeq 2000 (USA). Finally, hierarchical cluster analysis was performed on the differentially expressed miRNAs.

### Western blot

Extract the total protein of cells and exosomes, and use BCA assay to determine the protein concentration. Ten percent SDS-PAGE was used to separate the proteins. Then the proteins were transferred to PVDF membranes. The membranes were incubated with the primary antibody overnight at 4°C after blocked with 5%BSA. Subsequently, PBST was applied to wash the membranes. And at room temperature, the membranes were incubated with the secondary antibody. At last, proteins were visualized by an ECL Western blot detection system.

### Quantitative reverse transcription-polymerase chain reaction (qRT-PCR)

Extract a total of RNA of cells by TRIzol reagent (Invitrogen, USA). Then the RNA was converted into cDNA. Then qRT-PCR reaction was applied under the guidance of SYBR Green PCR Master Mix on a 7900HT Fast RT-PCR instrument (Takara, Japan) after the reverse transcription (RT) process. And the amplification procedure was as below: 95°C for 3 min, then 40 cycles at 95°C for 15 s, 60°C for 30 s. The relative expression of miRNA and mRNA were assessed by performing the 2^−∆∆Ct^ method. As controls, the U6 or GAPDH was applied, respectively.

### Cell transfection

A549 cells were purchased from ATCC (USA). In 6-well plates were 2 × 10^5^ cells/well seeded, and the cells were incubated at 37°C in a humidified atmosphere of 5% CO_2_. When cells grew to 50%–60%, miRNA-141 mimics and mimics NC were performed to transfect them with Lipofectamine 2000 reagent under the instructions of the manufacturer’s protocol. To each of the six-well plates 1 ml medium that contained 20% fetal bovine serum (FBS) was added at 6 h after transfection.

### Fluorescence assay

One hundred microliter of exosomes standby solution with concentration of 100 g/mL was resuspended in 1 mL PBS. Then add 4 μL PKH26 fluorescent dye solution and incubate at 37°C for 20 min. The mixture was centrifuged at 4°C at 100 000 × g for 70 min, and the supernatant was aspirated. And gently resuspended exosomes in 10 mL PBS. At 4°C, the exosomes were centrifuged at 100,000 × g for 70 min so as to separate the excess dye and discard the supernatant. Resuspended the exosomes in 100 μL PBS for later use. The fifth generation of HUVECs was resuspended in serum-free medium and placed in an incubator containing 5% CO_2_ at 37°C. After the cells were attached to the wall, the above PKH26 fluorescent labeled exosomes were added. After incubation for 12 h, wash the cells twice with PBS. And the cells were fixed with 4% paraformaldehyde and stained with DAPI. Confocal fluorescence microscopy was used to observe whether exosomes entered cells. With the help of a microscope, exosomes that were labeled with PKH26 demonstrated red fluorescence.

### CCK8

HUVECs in the logarithmic phase were digested and centrifuged and resuspended in serum-free medium. The cell density was altered to 1 × 10^4^/mL and each well was inoculated in a 96-well plate with 100 μL cell suspension. After cultured in an incubator for 24 h at 37°C and 5% CO_2_, the cells were completely adherent to the culture medium, and 90 μL serum-free medium was added in each well. Then, the cells were categorized into two groups: the experimental group added 10 μL miRNA-141 mimics exosomes with, and the control group added exosomes with the same amount of NC group. Five wells of each group were cultured for 1, 2, 3, 4, and 5 d, respectively. To each well 10 μL CCK-8 solution was added. Incubated the solution for 2 h. A multifunctional marker determined the absorbance value of each well at the wavelength of 450 nm.

### Transwell assay

A549 cells in the logarithmic phase were resuspended in serum-free medium and the cell concentration was controlled at 2 × 10^5^ cells/mL. Transwell upper chamber was inoculated with 200 μL cell suspension. Serum-free medium 500 μL and 100 g/mL miRNA-141 mimics exosomes 10 μL were added in the lower chamber of the experimental group, and serum-free medium 500 μL and NC group exosomes 10 μL were added in the lower chamber of the control group. After 24 h, the culture was stopped, in the upper chamber, the remaining cells were gently swabbed with sterile cotton swab, fixed for 20 min with 4% paraformaldehyde, the fixation solution was discarded, rinsed with distilled water for 2 times, and the dye solution was discarded, rinsed with distilled water and visualized under a microscope. Six random fields were taken from each group to count the cells passing through the membrane and take the mean value. The experiment was performed in tripcate.

### Wound healing assay

In terms of the wound healing assay, HUVECs were sown into 6-well plates and cultured for 48 h. And then with a new 10 μL pipette tip the cells were scratched across the center of the well, with PBS washed gently twice and refilled with fresh medium. The cells grew for an additional 24 h and were observed on a microscope.

### Tube formation assay

Matrigel matrix adhesive was placed in 96-well plate and incubated at 37°C for 30 min to solidify the Matrigel. HUVECs were resuspended in serum-free medium and inoculated into 96-well plates with 2 × 104 cells per well. The experiment was divided into two groups: 10 μL of miRNA-141 mimics exosomes containing 100 g/mL were added to the experimental group, and exosomes from the NC group were added to the control group. Each set has five multiple holes. After being treated at 37°C for 12 h, the tubular structures were visualized under an inverted phase contrast microscope, and the number of tubular structures per well was counted.

### Luciferase reporter assay

293 T Cells were cotransfected using Lipofectamine 2000 with either miRNA-141 mimics and pGL3‐GAX‐3′‐UTR or miRNA-141 mimics and pGL3‐GAX-MUT‐3′‐UTR. After 24 h, following cell collection, luciferase activities were measured using the Dual Luciferase kit (Promega, USA).

### RNA immunoprecipitation assay

According to previous reports, the MS2bs-MS2bp–based RNA immunoprecipitation (RIP) assay was put into effect. A549 cells were transfected, as control, with pcDNA-GAX-MS2, pcDNA-GAX-MS2, or pcDNA-MS2. After 48 hours, under the use of cells, RIP was performed by the use of a GFP antibody (Roach) and the Magna RIP RNA-Binding Protein Immunoprecipitation Kit (Millipore). Then qPCR analyzed the miRNA-141 level. A549 cells were transfected with miRNA-141 mimics or miRNA-141 control. After 48 hours, under the use of cells, RIP was performed by the use of an AGO2 antibody (Abcam) and then qPCR analyzed the GAX level. .

### Statistical analysis

Data were shown as mean ± SD from at the fewest three separate experiments. Statistical analysis was performed using the SPSS22.0 software. The Student t-test was applied to comparisons between groups. Differences in data were deemed statistically significant at P < 0.05.

## Results

### Extraction and identification of serum exosomes from lung cancer patients

**Figure 1.**
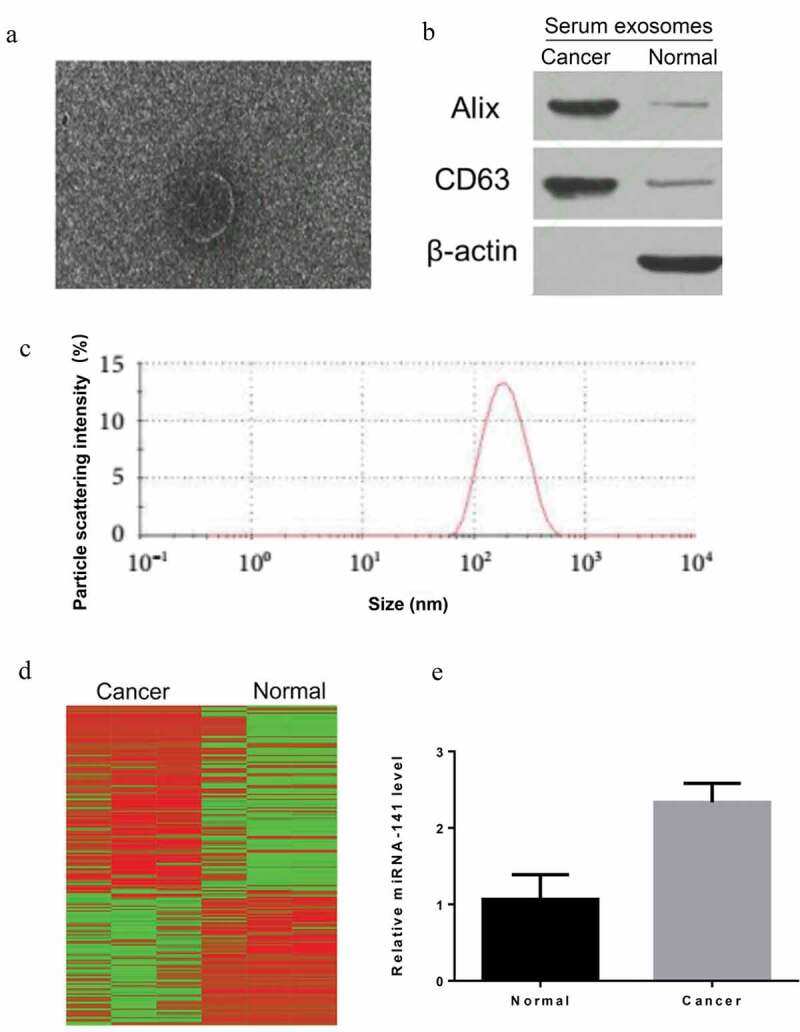
Extraction and identification of serum exosomes

In order to study exosome function secreted by lung cancer cells, we first extracted exosomes from serum by overspeed centrifugation method, and identified exosomes extracted by exosome markers Alix and CD63, which were verified by projection electron microscopy (Figure 1(a)) and Western blot (Figure 1(b)). The particle size range of exosomes detected by nanoparticle tracer and analyzer NanoSight was 30 ~ 200 nm (Figure 1(c)).

(a) The representative image of exosomes derived from serum exosomes from lung cancer patients analyzed by transmission electron microscopy. (b) The exosome markers Alix and CD63 in serum exosomes of lung cancer patients and normal people were detected by western blot. (c) Determination of particle size of exosomes by nanoparticle tracer and NanoSight instrument. (d) Analysis of miRNA expression profile of serum exosomes from lung cancer patients and normal controls by miRNA expression profile microarray and heatmap. (e) MiRNA-141 level was detected through qPCR.

### Screening of miRNA-141 in exosomes

miRNA expression profile microarray was first employed to measure the miRNA expression profile in serum exosomes of lung cancer patients and normal controls, and then heatmap analysis figured out a significant difference in the two populations (Figure 1(d)). By further integrating the chip results of the two groups, differential expression multiples, quantitative PCR and functional verification of bioinformatics analysis results, the significantly upregulated miRNA-141 (2.3 fold change) was concerned (Figure 1(e)).

### Exosome miRNA-141 promote the proliferation of endothelial cells

**Figure 2.**
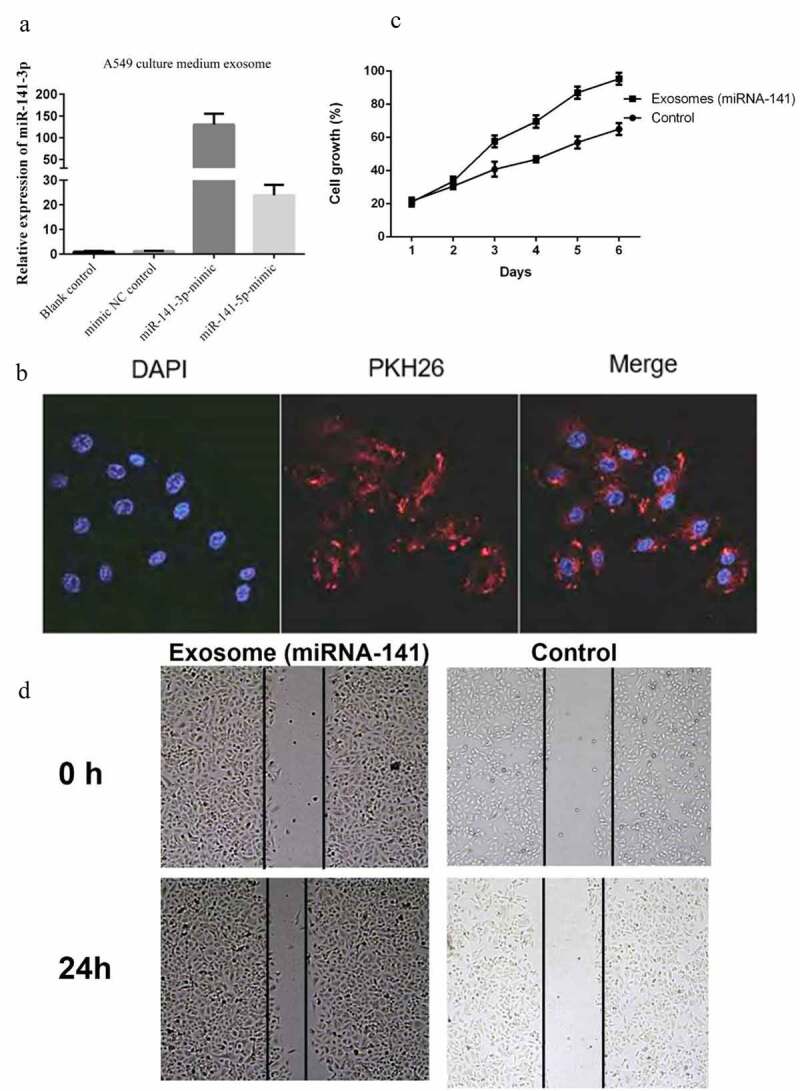
Exosome miRNA-141 promote lung cancer cells the proliferation and metastasis

miRNA-141 mimics were transfected in A549 cells, and then exosomes were extracted to detect the level of miRNA-141. It was observed the marked elevation of both the miRNA-141-3p and miRNA-141-5p compared with that of the control groups (Figure 2(a)). These exosomes were extracted and stain by membrane dye PKH26, then co-cultured with HUVECs, it was observed under confocal microscopy that exosomes were absorbed by HUVECs and distributed around the nucleus, indicating that exosomes could enter HUVECs (Figure 2(b)). We found that exosomes co-cultured with HUVECs could significantly promote the cell proliferation (Figure 2(c)). The results of the scratch test showed that the addition of exosome miRNA-141 could significantly promote the scratch healing ability of HUVECs (Figure 2(d)).

(a) MiRNA-141 was overexpressed in A549 lung cancer cells through transfection, and exosomes were extracted to detect the level of miRNA-141. (b) These exosomes were extracted and stain by membrane dye PKH26, then co-cultured with HUVECs, it was observed under confocal microscopy that exosomes were absorbed by HUVECs and distributed around the nucleus. (c) We found that exosomes co-cultured with HUVECs could significantly promote the cell proliferation. (d) The results of the wound healing assay showed that the addition of exosome miRNA-141 could significantly promote the scratch healing ability of HUVECs.

### miRNA-141 promote the lung cancer cell metastasis and endothelial cells tubular structure formation

**Figure 3.**
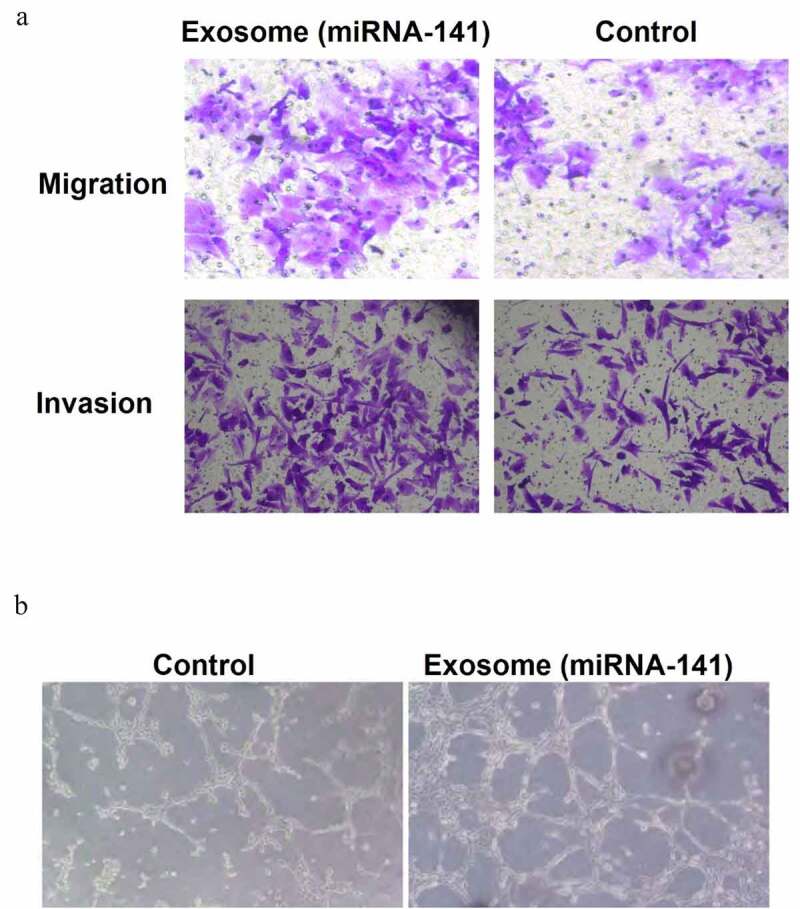
miRNA-141 promote the lung cancer cell metastasis and endothelial cells tubular structure formation

miRNA-141 was overexpressed in A549 cells through transfection. Transwell migration assay showed a marked increase of the number of transmemmal migration cells in the exosomal miRNA-141 group as compared to the control group, indicated the promotion of lung cancer cell migration and invasion by miRNA-141 (Figure 3(a)). The observation under inverted phase contrast microscope showed that only non-closed polygonal structures were formed in the control group, while more tubular structures were formed in the exosome miRNA-141 group. As compared to the control group, the tubular structure of the experimental group was faster and more complex. The number of tube samples in the experimental group was (76.0 ± 5.0)/hole, which was significantly higher than that in the control group (36.0 ± 2.6)/hole, with a significant difference between the two (T = 15.910, P = 0.001), indicating that miRNA-141 promote the formation of tubular structure (Figure 3(b)).

(a) miRNA-141 was overexpressed in A549 cells through transfection. Transwell migration assay showed a marked increase of the number of transmemmal migration cells in the exosomal miRNA-141 group as compared to the control group. (b) Observation under an inverted phase-contrast microscope showed that only non-closed polygonal structures were formed in the control group, while more tubular structures were formed in the exosome miRNA-141 group. As compared to the control group, the formation of tubular structure of the experimental group was faster and more complex, and the number of tube samples (76.0 ± 5.0/hole) in the experimental group was significantly higher than the (36.0 ± 2.6)/hole in the control group.

### miRNA-141 downregulated GAX expression

**Figure 4.**
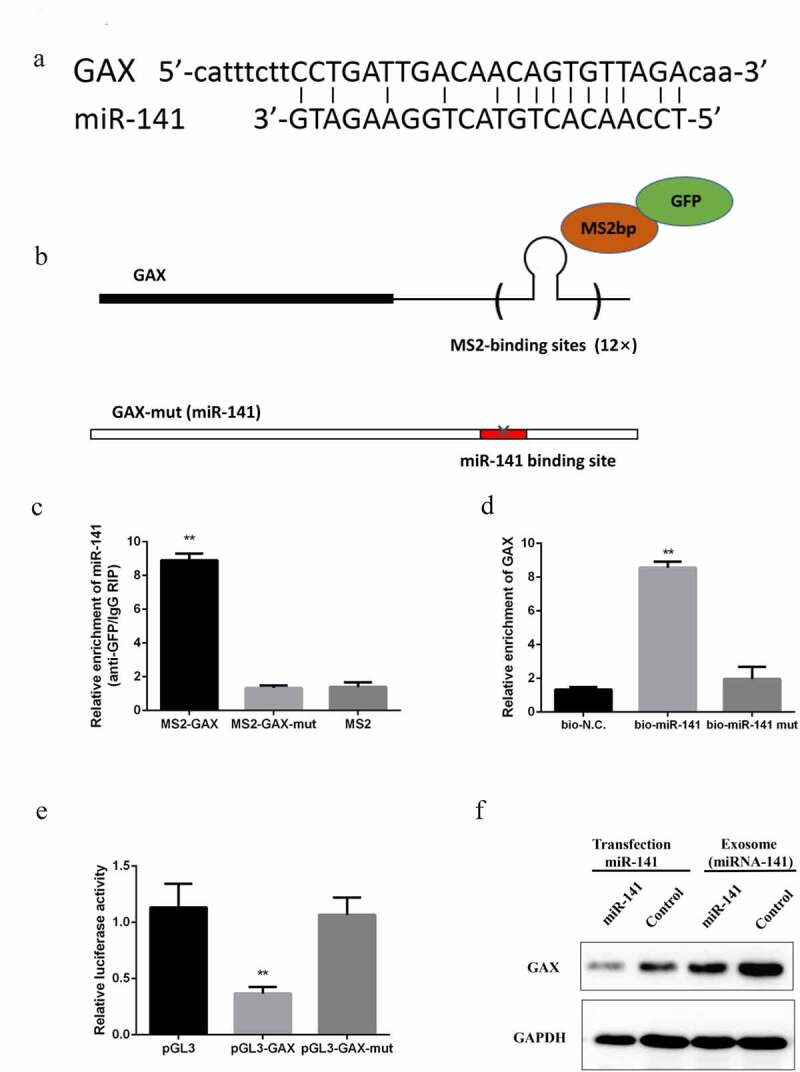
miRNA-141 downregulates GAX expression

In an attempt to explore the mechanism of miRNA-141 regulated tumor metastasis and angiogenesis, bioinformatics analysis was first performed and found a binding site of GAX and miRNA-141 (Figure 4(a)). The direct binding of the two at endogenous was further validated by MS2-binding protein (MS2bp)-based RIP, with a specific binding between MS2bp and RNA containing MS2-binding sequences (MS2bs). Subsequently, A549 cells were transfected by a generated construct containing GAX transcripts combined with MS2bs elements (Figure 4(b)) and a construct containing MS2bp-GFP. By using GFR antibody and IgG as a negative control, immunoprecipitation was carried out, followed by qPCR-based detection of miRNA-141. The result showed a marked enrichment of miRNA-141 by GAX as compared to the empty vector (MS2) and MS2-GAX-MUT (Figure 4(c)). By using a biotin-labeled GAX probe, RNA pulldown assay for further validation of the specific combination between GAX and miRNA-141 was performed. In addition, an inverse pulldown assay was confirmed that miRNA-141 could pulldown GAX (Figure 4(d)). Luciferase assay based on a luciferase construct containing GAX or GAX-MUT, showed a marked inhibition of luciferase activity of GAX by miRNA-141, while little effect of it on GAX-MUT (Figure 4(e)). We detected GAX expression levels in HUVECs after co-cultured with exosome miRNA-141, we found that GAX were significantly downregulated (Figure 4(f)).

(a) Binding site of miRNA-141 to GAX. (b) A construct containing GAX transcripts combined with MS2bs elements. (c) After MS2-RIP, qPCR analysis was performed to determine miRNA-141 expression. (d) Incubation of A549 cell lysates with biotin-labeled GAX and qPCR-based determination of miRNA-141 expression after pulldown. (e) Luciferase activity in A549 cells co-transfected with miRNA-141 and luciferase reporter containing GAX or GAX-MUT, with results presented as the relative ratio of firefly to Renilla luciferase activity. (f) The detection of GAX expression levels in HUVECs after co-cultured with exosome miRNA-141.

## Discussion

The maintenance of tumor microenvironment by paracrine signaling between tumor and surrounding stromal cells plays a pivotal role in lung cancer progression. By shuttling functional RNAs, exisomes as nanovesicles secreted actively by tumor cells functions as mediators in intercellular communication. In addition, cell-cell communication can also be mediated by miRNAs. However, the specific mechanism by which exosomal miRNAs function in lung cancer angiogenesis is currently unknown.

In recent years, miRNAs in exosomes began to attract people’s attention. When Kogure’s team was studying exosomes from liver cancer cells, it was firstly observed an increase of expression of several ultra-conservative miRNAs in exosomes [21]. And exosomes would be consumed by the surrounding cancer cells after being released, correspondingly contributing to the promotion of cancer cell proliferation [22]. MiRNAs in exosomes secreted by drug-resistant renal carcinoma cells function in the increase of resistance to sunitinib in sensitive renal carcinoma cells by transporting into these sensitive cells [23]. Endothelial cells derived exosome miR-126 modulates adhesive and migratory abilities of chronic myelogenous leukemia cells [24]. Collectively, current studies have suggested the functions of miRNAs carried by exosome vector in the proliferation, metastasis and drug resistance of cancer cells.

Herein, our study focused on determining whether miRNAs participate in the malignant progression of lung cancer. For this purpose, miRNA expression profile microarray was first employed to measure the miRNA expression profile in serum exosomes of lung cancer patients and normal controls, and then heatmap analysis figured out a significant difference in the two populations. By further integrating the chip results of the two groups, differential expression multiples, quantitative PCR and functional verification of bioinformatics analysis results, the significantly upregulated miRNA-141 was concerned. MiRNA-141 belonging to miR-200 family regulates a series of biological processes under physiological and pathological conditions by binding to different targets and regulating different signaling pathways, especially in aspects such as angiogenesis and tumor. The formation and development of vascular system is a complex process, which is maintained by the dynamic balance between promoting angiogenesis and anti-angiogenesis proteins. Recent studies have shown that miRNA appears as a key regulator of many cellular processes, including angiogenesis [25]. In the vascular system, miRNAs can not only participate in the process of promoting angiogenesis or enhancing angiogenesis, but also play an anti-angiogenesis role by promoting endothelial cell apoptosis and inhibiting endothelial cell proliferation [13,26].

Further verified by in vitro functional experiments based on co-culture of lung cancer exosomes and HUVECs, lung cancer exosomes could enter the HUVECs cytoplasm. Moreover, in vitro experiments showed that the exosomes with high expression of miRNA-141 promoted the proliferation, migration and tubular differentiation of HUVECs. Further bioinformatics analysis revealed that miRNA-141 targeted the binding of GAX to the 3ʹUTR region, suggesting that miRNA-141 may directly transcribe the expression of GAX. GAX is homologous box genes, which are expressed in vascular smooth muscle cells and endothelial cells, and their high expression can inhibit angiogenesis [27]. All the above results suggest the involvement of lung cancer cell-derived exosomal miRNA-141 in the angiogenesis of lung cancer tissues.

## Conclusion

In summary, we herein demonstrated that miRNA-141, which is highly expressed in lung cancer cells, enters tumor stromal vascular endothelial cells through exosome pathway. MiRNA-141 promotes angiogenesis and promotes malignant progression of tumors by targeting the expression of GAX.

## Supplementary Material

Supplemental Material

## Highlights

1. miRNA-141 was up-regulated in the lung cancer serum exosomes.
2. Tumor-derived exosomal miRNA-141 promoted A549 cell proliferation and metastasis.
3. Tumor-derived exosomal miRNA-141 promoted angiogenesis.
4. GAX is a target gene of miRNA-141.
