# Supplementary figures and images for: Tumor-derived exosomal miRNA-141 promote angiogenesis and malignant progression of lung cancer by targeting growth arrest-specific homeobox gene (GAX)
Source: Bioengineered. 2021 Feb 25;12(1):821–31. doi: 10.1080/21655979.2021.1886771 (PMC8291845; doi:10.1080/21655979.2021.1886771)

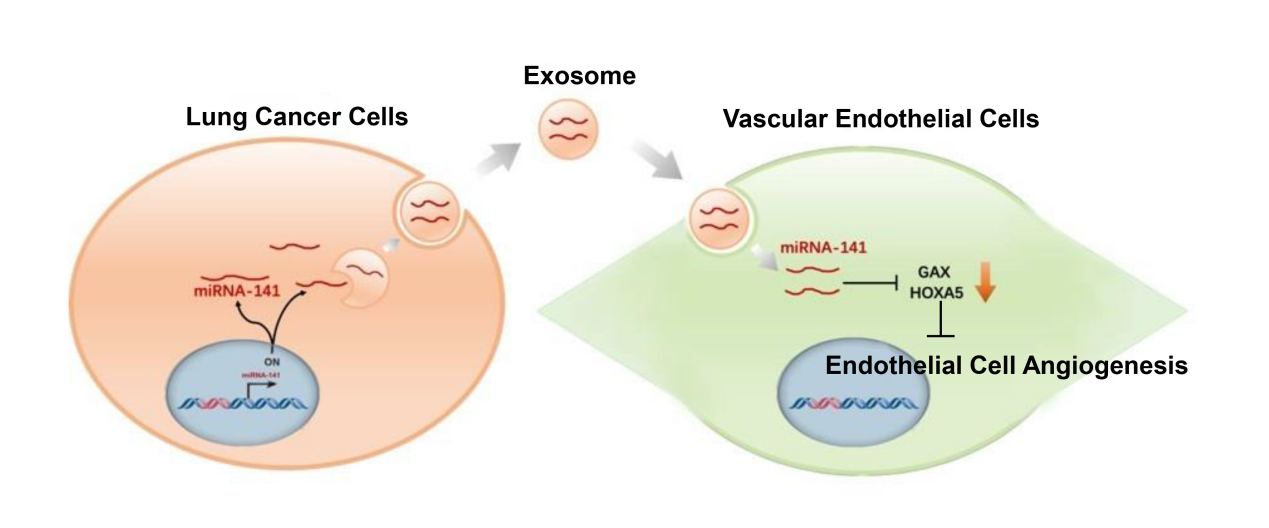

Supplement: Supplemental Material [file KBIE_A_1886771_SM3352.tif]
